# An evaluation of paediatric tinnitus services in UK National Health Service audiology departments

**DOI:** 10.1186/s12913-020-5040-y

**Published:** 2020-03-14

**Authors:** Harriet Smith, Kathryn Fackrell, Veronica Kennedy, Johanna G. Barry, Emily Broomhead, Derek J. Hoare

**Affiliations:** 1 NIHR Nottingham Biomedical Research Centre, Hearing Sciences, Division of Clinical Neuroscience, School of Medicine, University of Nottingham, Nottingham, UK; 2 National Institute for Health Research, Evaluation, Trials and Studies Coordinating Centre (NETSCC), University of Southampton, Southampton, UK; 3 Bolton NHS Foundation Trust, Greater Manchester, UK; 4 Nottingham University Hospitals NHS Trust, Nottingham, UK; 5 British Tinnitus Association, Sheffield, UK

**Keywords:** Tinnitus, Child, Audiology, Care provision, Clinical management, Healthcare quality, Service evaluation

## Abstract

**Background:**

Whilst evidence indicates many children experience troublesome tinnitus, specialist services for children are far less established than those available for adults. To date, there is limited understanding of how paediatric tinnitus is managed in the UK, and to what extent current practice reflects what is recommended. This service evaluation aimed to 1) profile how tinnitus in children is managed in UK clinical practice, and assess to what extent care provided by services reflects advice included in the British Society of Audiology (BSA) Tinnitus in Children Practice Guidance, 2) collate clinician opinions on how services may be optimised, and 3) identify common problems experienced by children who present with bothersome tinnitus in clinic.

**Methods:**

As part of a larger survey, eight questions regarding services for paediatric tinnitus were distributed to UK NHS audiology services via email and social media. Representatives from eighty-seven services responded between July and September 2017.

**Results:**

Fifty-three percent of respondents reported that their department provided a paediatric tinnitus service. Among these services, there was widespread use of most BSA recommended assessments and treatments. Less widely used practices were the assessment of mental health (42%), and the use of psychological treatment approaches; cognitive behavioural therapy (CBT) (28%), mindfulness (28%), and narrative therapy (14%). There was varied use of measurement tools to assess tinnitus in children, and a minority of respondents reported using adult tinnitus questionnaires with children. Frequently reported tinnitus-related problems presented by children were sleep difficulties, concentration difficulties at school, situation-specific concentration difficulties, and emotional distress.

**Conclusions:**

Approaches used to manage children with troublesome tinnitus in UK NHS services are largely consistent and reflective of the current practice guidance. However, findings from this study indicate specialist staff training, access to child-specific tools, and the treatment and referral of children with tinnitus-related psychological problems represent key areas in need of optimisation.

## Background

Tinnitus is the perception of sound in the ears or head without any external source. For some, this symptom can be troublesome, causing problems in many different areas of life, such as with sleep, psychological health, or cognitive performance [1, 2]. Those with troublesome tinnitus may benefit from referral to specialist health services for assessment and treatment support [3]. Prevalence data suggest that similar proportions of adults and children experience troublesome tinnitus; Davis [4] reported 2.8% of adults to have tinnitus that was “moderately annoying or worse”, and similarly, Humphriss et al. [5] reported 3.1% of children to experience “clinically significant” tinnitus. Despite this, tinnitus in children is a relatively unrecognised problem and consequently, tinnitus treatments and health services for children are far less established versus those available for adults [6].

In the UK, the majority of tinnitus services exist in audiology departments, with over 140 UK audiology departments providing an adult tinnitus service [7, 8]. In contrast, tinnitus services for children are sparse [6]. Both UK-based and international research has indicated that very few children with tinnitus access specialist care [6, 9–11], and it is common for children to be managed within adult services rather than child-specific services [6, 12]. The need to optimise clinical management of children with tinnitus was highlighted by members of the public and clinicians in a 2011/2012 priority setting consultation exercise [13]. To address this need, the 2015 BSA “Tinnitus in Children Practice Guidance” [14] was developed, offering a toolkit of child-friendly clinical management strategies to support paediatric service providers. Whilst the guidance was based on the authors’ clinical experience and research evidence available at the time [15], the authors highlight the lack of robust scientific evidence to support their assessment and treatment recommendations. To date, there is limited understanding of how paediatric tinnitus is managed in the UK, and the extent to which current practice reflects the recommended approaches described in the BSA Guidance.

The importance of health services tailored to the needs of children is well recognised in hearing health and more broadly across other disease areas [16, 17]. Children differ from adults in terms of their physical, emotional, and intellectual development, and this has implications for their treatment needs, both in terms of the treatment they receive and the environment in which they are cared for. When providing care, the needs of the child’s family must also be carefully managed. The BSA Guidance supports the provision of child-specific tinnitus services. Whilst the tinnitus ‘sound’ may be experienced similarly by children and adults, research has highlighted key differences in how children and adults present at clinic, and these differences have important implications for the provision of appropriate care. For example, whilst several studies have found children and adults often use similar words to describe the sound of tinnitus (e.g. “ringing” or “buzzing”) [18, 19], it is also common for children to use emotive or creative descriptors (e.g. “angry bees” buzzing) [14], or to form narratives to help them make sense of their experience (e.g. belief tinnitus is caused by “a monster in their head”) [20]. Furthermore, several studies have shown children to rarely spontaneously discuss their tinnitus with adults [19, 21], which may mean they are less forthcoming when discussing tinnitus with a health care provider. These linguistic and communication differences highlight the challenges of reliable tinnitus assessment and measurement of treatment benefit in children, underlining the importance of skilled practitioners and child-friendly approaches. The BSA Guidance advises that adult models of tinnitus management should be adapted for use with children, ensuring that children’s communication, development, and linguistic needs are catered to. The use of play, drawing and visual or simplified methods of communication are suggested as helpful techniques [14].

Studies have found children to report some of the same tinnitus-related problems commonly experienced by adults [22]. However, whilst the problems experienced by adults are well documented [2, 23, 24], there is limited understanding of the scope and impact of tinnitus-related problems experienced by, and important to, children. Knowledge of tinnitus problems in adults has guided and improved clinical assessment and treatment practices, e.g. directly informing the design of clinical questionnaire measures of tinnitus impact, and supporting clinicians in their assessment of tinnitus severity and measurement of treatment-related change [25–27]. Several tinnitus questionnaire measures are available for use with adults and are sensitive to the tinnitus-related problems they experience, however, none have been designed for use with children [14].

The purpose of this service evaluation was to 1) profile how tinnitus in children is managed in UK clinical practice, and assess to what extent care provided by services reflects advice included within BSA Guidance, 2) collate clinician opinions on how services may be optimised, and 3) identify common problems experienced by children who present with bothersome tinnitus in clinic.

## Methods

This study was a part of a British Tinnitus Association (BTA) service evaluation of tinnitus services in UK National Health Service audiology departments. The evaluation is carried out annually or bi-annually and is conducted as an online survey containing about 50 items. The primary objective of the evaluation was to generate a database of up-to-date information about the tinnitus services available throughout the UK. The main survey is a consistent set of questions used every time the survey is conducted. With each issue, a small set of questions are added to capture more detailed information about one aspect of the service. In 2017, this involved eight questions about paediatric tinnitus services (Appendix 1). The data from these eight questions were analysed here. Data were analysed with the support and permission of the data controller (EB). This use of the data complies with the governance procedures of the charity. As this survey only used data for the purpose of service evaluation, individual consent was not sought, and research ethics committee review was not required [28]. Survey respondents were clinical professionals and minimal personal data was collected. This service evaluation is reported according to Checklist for Reporting Results of Internet E-Surveys (CHERRIES) [29].

### Questionnaire development

The eight questions on paediatric tinnitus were designed via an iterative process. Initial questions were drafted to assess key aspects of paediatric tinnitus services. Questions were further informed by assessment and treatment practices described in the BSA Guidance, with input from expert clinicians in the field. Questions were first drafted by DJH, and then appraised by EB. A test version of the survey was reviewed before the study was launched. There was a mix of closed questions (dichotomous and multiple response) and open questions. Multi-choice questions included an extensive list of answer options in addition to an ‘other (please specify)’ free text option. Closed questions asked about whether the department offered a paediatric tinnitus service, the clinical roles of those responsible for managing children with tinnitus, assessment procedures used, measurement tools used, and treatments offered. Open questions asked about common problems reported or identified in children with tinnitus, the percentage of children referred to Child and Adolescent Mental Health Services (CAMHS), and requested suggestions regarding how the paediatric tinnitus service could be improved. Responses to each question were optional. The survey was uploaded onto surveymonkey.com with 1–2 questions per page, and logic to omit irrelevant questions. Participants could revisit and edit any of their responses up until the point they chose to submit (on the final page).

### Distribution

The service evaluation was delivered online via surveymonkey.com from 17th July 2017. A link to complete the survey was sent out via email to all contacts registered on the BTA database of UK-based audiologists and National Health Service (NHS) audiology departments (approximately 200). The link was also shared on the BTA’s social media channels. At the time of invitation, respondents were informed about the objective and length of the survey and were informed that, upon survey completion, participants would be sent two complimentary copies of the charity’s quarterly magazine. Responses were obtained between July 2017 and September 2017.

### Data collection and analysis

A single representative was asked to complete the questionnaire on behalf of each service. Where duplicate responses from the same department were found, services were contacted to determine which data set was most representative of the service; that dataset was used in analyses. All queries were handled via the data controller (EB). Data from closed questions were analysed in Microsoft Excel and presented as the number and percentage of respondents from the total number answering each question. Qualitative data from open-ended questions were grouped by topic by HS and DJH. An inductive, ‘bottom-up’ approach was used to group responses into topics and sub-topics [30]. Participant quotes are used in this report to illustrate the sub-topics identified.

## Results

Eighty-seven participants responded to the survey, representing 87 individual UK NHS audiology departments. Of these, 46 (53%) reported that their department provided a paediatric tinnitus service. Most commonly, audiologists (including senior and specialist audiologists) were reported as responsible for the management of children with tinnitus (80%, *n* = 35). Hearing therapists (39%, *n* = 17), ENT specialists (39%, *n* = 17), audio-vestibular physicians (16%, *n* = 7), clinical psychologists (7%, *n* = 3), and clinical scientists (7%, *n* = 3) were also involved. One respondent reported that a *“paediatrician specialising inaudiology”* was involved in the management of tinnitus in children at their service.

### Management of tinnitus in children/ use of BSA recommended approaches

**Fig. 1:**
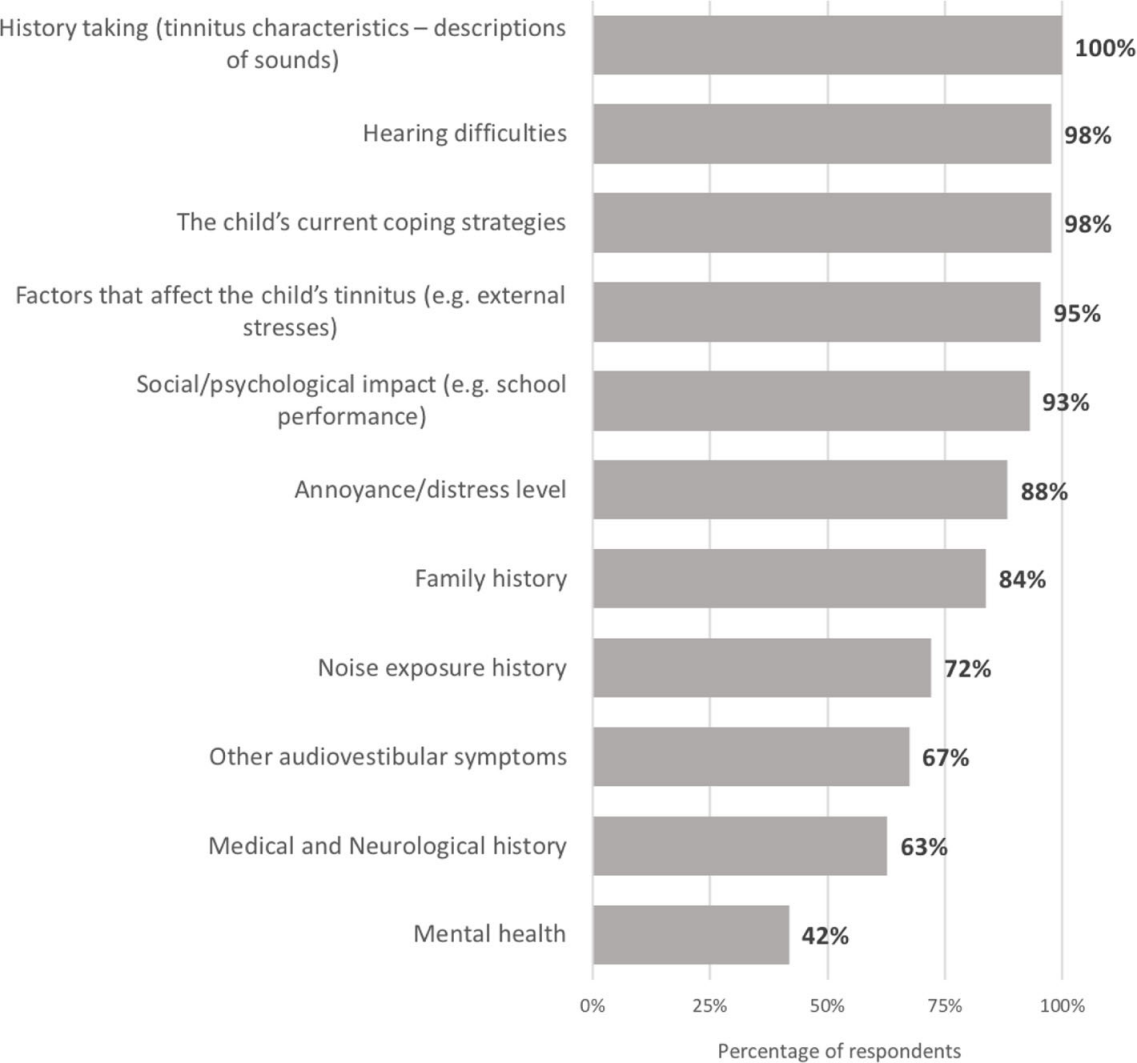
Use of BSA recommended assessment approaches for tinnitus in children (*n*=43)

Figure 1 shows the elements that services included in their assessment of children with tinnitus. Most services assessed tinnitus history (tinnitus characteristics – descriptions of sounds) (100%, *n* = 43), hearing difficulties (98%, *n* = 42), current coping strategies (98%, *n* = 42), and factors that affect the child’s tinnitus (e.g. external stresses) (95%, *n* = 41). Fewer services assessed mental health (42%, *n* = 18).

When asked about the use of measurement tools to assess tinnitus-related problems in children, 35% (*n* = 15) did not report the use of any measurement tools. Others reported the use of visual analogue scales (37%, *n* = 16), self-devised questionnaire measures (26%, *n* = 11), Likert scales (12%, *n* = 5), adult tinnitus questionnaires (12%, *n* = 5), and/or paediatric questionnaires relating to psychological health or education (9%, *n* = 4). Adult questionnaires used were the Tinnitus Handicap Inventory (THI) [26], the Tinnitus Functional Index (TFI) [27], and the Mini-Tinnitus Questionnaire (Mini-TQ) [31]. Of the five respondents who reported the use of adult questionnaires, two reported that use was limited to older children. Paediatric questionnaires used were the Revised Children’s Anxiety and Depression Scale (RCADS) [32], the Paediatric Index of Emotional Distress Questionnaire (PI-ED) [33], the Strengths and Difficulties Questionnaire (SDQ) [34], and questionnaires for educational assessment. It is unknown if parent and/or child versions of the RCADS or SDQ were used. One respondent reported the use of a *“0-10 scale for the parent to rate how intrusive the tinnitus appears to be”* when assessing very young children. Another respondent reported use of a *“children’s anxiety index scale”*.

**Fig. 2:**
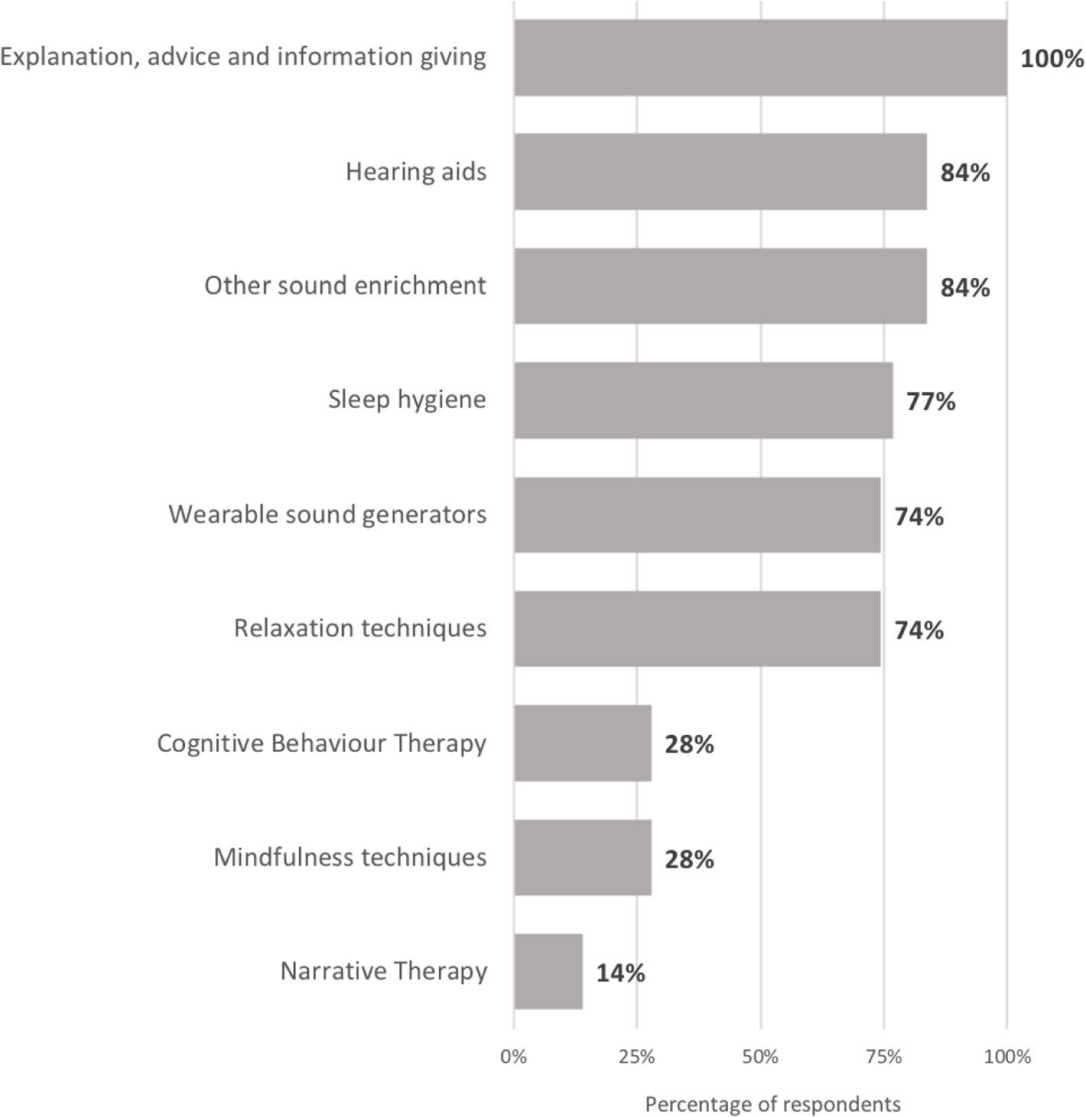
Use of BSA recommended treatment approaches for tinnitus in children (*n*=43)

Figure 2 shows the tinnitus treatment approaches used by paediatric services. The general therapeutic approach of ‘explanation’ and ‘advice giving’ was used most widely (100%, *n* = 43). There was also widespread use of hearing aids (84%, *n* = 36) and non-wearable sound enrichment (84%, *n* = 36). Less commonly used approaches were those addressing psychological problems (i.e. narrative therapy [14%, *n* = 6], CBT [28%, *n* = 12], and mindfulness techniques [28%, *n* = 12]).

Child and Adolescent Mental Health Services (CAMHS) refers to the UK NHS services responsible for working with children and young people who have difficulties with their emotional or behavioural wellbeing. The BSA Guidance advises that referral to CAMHS should be considered for children who show signs of significant psychological distress such as anxiety or depression, and would therefore require help from a trained mental health practitioner [14]. When asked about the percentage of children who require referral to CAMHS, respondents reported a range between 0 and 50%, with most services referring ≤20% of children. Several respondents reported that although few children were referred from their service to CAMHS, many were already under CAMHS, or had been referred by CAMHS to their service.

### Clinician opinions on how services may be optimised

Table 1 reports data from an open-ended question whereby respondents were asked to suggest ways in which their paediatric tinnitus service could be improved. The most common suggestions called for improved connections with mental health/paediatric services, more child-friendly resources, and more general training in the area of paediatric tinnitus. There were single suggestions regarding improved staff expertise and access to therapeutic devices.

**Table 1:** Clinician opinions on how services could be optimised: topics, sub-topics, and example quotes. N refers to the number of respondents who made a suggestion relating to the sub-topic

Topics	Sub-topics	n	Example Quotes
More experience	More experience/time to develop services	3	*“Experience, this is a new service”* *“This service is new to the department so there are many improvements that will be made as the service is developed”* *“More experience/confidence”*
	More patients	3	*“We need greater exposure to children with tinnitus in order to gain greater competency in dealing and talking through tinnitus with children and families”* *“Encourage ENT to ask more children if they hear noises in their ears. More patients will lead to more experience”* *“Only a few children are referred”*
Improved organisation	Improved organisation (general)	2	*“More coherent strategy”* *“An organised approach”*
	Improved scheduling	2	*“More time to book appointments close together. To see children in a separate clinic - it is difficult to see them when they are mixed into adult clinics”* *“Shorter waiting times”*
	Funding/ commissioning	3	*“At the moment there is no commissioned service so it is very* ad hoc*”* *“More funding for staff and time”*
	Improved connections with mental health /paediatric services	8	*“We hope to work on improving our network with local mental health professionals”* *“Direct Audiology links with CAMHS”* *“Access to paediatric CBT”* *“Direct access to relaxation/anxiety/stress management”* *“Confidence that CAMHS services understand tinnitus and how this can be managed as part of a wider mental health support plan”*
	Access to therapeutic devices	1	*“Access to provide patient with supportive devices or offer direct purchase”*
Child-friendly resources and environment	Child-friendly resources (general)	5	*“More child-friendly resources”* *“Interactive material”* *“More visual tools for explanation for young children”* *“A teen leaflet”*
	Child-friendly assessment resources	2	*“Some good history sheets aimed at younger children…at the child’s level of understanding”* *“Child-friendly tinnitus questionnaire to help outcome measures”*
	Child-friendly environment	3	*“Child-friendly counselling room”* *“Child-friendly environment - more toys/posters/playmats etc”*
Staff improvements	Training (general)	4	*“Staff to attend the annual paediatric training course”* *“Training specific to paediatrics”* *“Need more staff trained in this area”*
	Training in delivery of psychological therapies	3	*“Learning how to teach relaxation and mindfulness techniques”* *“More staff trained in all aspects of behavioural therapies or a referral route for this”*
	Improved staff knowledge/ interest	2	*“Improving interest on the subject within the team”* *“Networking with other clinicians would be very useful”*
	Additional staff expertise	1	*“Having a child psychologist on board”*

### Common problems experienced by children with bothersome tinnitus

Respondents reported a variety of common problems they encountered in children with bothersome tinnitus (Table 2). Most frequently reported topics were sleep difficulties, concentration difficulties at school, situation-specific concentration difficulties, and emotional distress. Less frequently reported topics were lack of support from others, fatigue, behaviour problems, and worries.

**Table 2:** Problems reported as common in children with bothersome tinnitus: topics, sub-topics, and example quotes. N refers to the number of respondents who mentioned problems relating to the sub-topic

Topics	Sub-topics	n	Example Quotes
Physical Health	Sleep difficulties	24	*“Sleep* [difficulties and] *hearing noises at night”* *“Struggling to get to sleep”* *“Not sleeping well”*
	Fatigue	1	*“Feeling tired”*
Concentration difficulties	General concentration difficulties	6	*“Concentration/attention issues.”* *“Finds tinnitus distracting: impedes ability to concentrate”*
	Concentration difficulties at school	11	*“Affects concentration, especially at school”* *“Fear* [that] *difficulty concentrating will affect performance in the classroom”*
	Situation-specific concentration difficulties (in noisy, quiet, during exams, or when studying)	10	*“Difficulty concentrating at school during exams and/or in a quiet classroom”* *“Older children - difficulty concentrating in exams”* *“Finding it difficult to concentrate when studying”*
Hearing difficulties	General hearing difficulties	3	*“Hearing Loss”* *“Difficulties with listening”*
	Hearing difficulties at school	3	*“Difficulty hearing the teachers voice”* *“*[Finding it] *difficult to hear in class or what friends are saying”*
Emotional difficulties	Distress (e.g. anxiety, panic, stress)	7	*“General anxiety or panic symptoms”* *“Anxiety related to tinnitus”*
	Low mood (e.g. depression, reduced enjoyment)	3	*“Sometimes low mood”* *“*[Difficulty] *enjoying quiet time”*
	Worries	2	*“Worry about* [tinnitus] *getting worse or something is seriously wrong”*
	Behaviour	1	*“Behavioural affects”*
Lack of understanding of tinnitus	n/a	3	*“Not knowing what it is”* *“Anxiety regarding what the noises are”* *“Not having a rational explanation and reassurance”*
Lack of support from others	n/a	1	*“Other people don’t believe it, or the extent of it, or why it causes the distress it does”*

## Discussion

This service evaluation assessed how tinnitus in children is currently managed in UK clinical practice, collated clinician opinions on how services may be optimised, and identified a variety of common problems experienced by children who present with bothersome tinnitus in clinic.

### Management of children in UK clinical practice

Assessment of children in UK paediatric tinnitus services largely reflects the recommended procedures described in the BSA Guidance [14]. Whilst the child’s ‘mental health’ was the least commonly assessed factor, the majority of respondents reported that their service assessed the ‘social/psychological impact of tinnitus’, and the child’s ‘annoyance/distress level’. Given several respondents reported the common presentation of children with tinnitus-related emotional problems, assessment of these factors is highly relevant. The BSA Guidance advises that paediatric questionnaire measures of psychological health can be helpful in providing a formal assessment of the child’s mental health. These measures can not only be useful in understanding the impact of tinnitus on the child’s emotional wellbeing, but can also screen for significant psychological distress. Data collected may also help to facilitate the referral of a child to mental health services when necessary. It may therefore be useful for paediatric services to include this assessment approach, in appropriate cases, where they are not already doing so. Unsurprisingly, given the lack of a child-specific tinnitus questionnaire, this evaluation found limited and inconsistent use of measurement tools to assess children. Amongst those that used tools, a small minority reported the use of adult tinnitus questionnaires when assessing children. The formal use of adult tinnitus questionnaires with children is not appropriate given that instruments are likely to be overly complex and burdensome for children to complete [35] and are unlikely to have relevance to children in terms of assessing the tinnitus-related problems that are important to them. These findings suggest that the assessment of tinnitus in children may benefit from the provision of standardised, child-specific measures. A suggestion from a respondent in this study supported this idea, stating that a *“child-friendly tinnitus questionnaire” *would help to improve their service.

This evaluation found that the treatment provided by UK paediatric tinnitus services was largely reflective of the approaches described in the BSA Guidance. However, despite tinnitus-related emotional difficulties being reported as common in children presenting in clinic, only a minority of services used psychological therapies. The BSA Guidance advises that psychological techniques can be helpful in giving children strategies to overcome tinnitus-related emotional difficulties, and often practitioners within paediatric audiology services can deliver these strategies. The infrequent use of narrative therapy, CBT, or mindfulness, in addition to respondents’ suggestions for more training in this area, indicate a lack of confidence amongst clinicians in delivering these approaches. Furthermore, varied rates of referral to CAMHS, in addition to calls for improved connections with mental health services, suggests paediatric services are limited in their ability to refer children with significant psychological distress. Together, these findings suggest that the provision of appropriate and accessible psychological treatment represents a service area in need of development. Similarly, a 2012 evaluation of UK NHS adult audiology services found less than half of adult tinnitus services offer psychological interventions [36]. To address this unmet need, work is ongoing in the UK to support the inclusion of psychological support for adults with tinnitus into routine audiologist practice [37].

This evaluation found just over half of UK audiology departments offer a paediatric tinnitus service, indicating there are significantly fewer services available for children than are available for adults. Several respondents suggested that their paediatric tinnitus service received very few referrals, and that more patients and experience are needed in order to establish and improve their service. This reflects other UK and international studies in this field, where very few children have been reported to have accessed treatment [9–11]. Whilst small numbers of children accessing care could suggest a limited demand for paediatric tinnitus services, this idea contrasts with findings from prevalence studies that have indicated significant numbers of children experience troublesome tinnitus. Limitations in children’s ability to report their tinnitus-related problems may explain this discrepancy. Unlike adults, children communicate their health problems via an adult gatekeeper (e.g. a parent) in order to seek help. As stated previously, several studies have found that children rarely report tinnitus spontaneously to adults [19, 21]. Yet when asked directly, more children will report their tinnitus [19, 21, 38]. Age-related cognitive and linguistic limitations could play a role, restricting children’s ability to communicate their symptoms clearly [39]. Furthermore, it is also possible that, when children raise concerns about their symptoms, their complaints are not being recognised as significant. A general lack of public awareness that tinnitus can affect and cause problems in children may contribute to this issue [40]. These factors represent barriers to parents becoming aware of the child’s tinnitus, recognising it as a significant problem, and accessing care for their child.

Barriers to children accessing care may also exist within the healthcare system. Sometimes tinnitus in children may be overlooked because clinicians lack awareness, confidence, or training in how to manage it. A study in Finland by Szibor et al. [11] found an average 12 month delay between children first reporting tinnitus symptoms and their presentation at a specialist tinnitus clinic, suggesting significant delays between children visiting local services and their referral to a specialist. Surveys of UK clinicians managing tinnitus in children conducted in 2009 and 2012 by Kennedy et al. [6] evidenced a need for more child-specific training on tinnitus management. These surveys also found some clinicians were reluctant to discuss tinnitus with children due to an unfounded fear that it could draw the child’s attention to tinnitus and cause unnecessary distress. The need for more child-specific training, staff expertise, and support networks, was also reported by respondents in the present study. In the UK, there are two professional courses that offer child-specific training on tinnitus management; the University College London *“Tinnitus and Hyperacusis in Adults and Children: Mechanisms, Assessment and Management Masterclass”* [41] and the BTA *“Assessment and Management of Tinnitus in Children”* course [42]. Adults attending audiology services are routinely asked about tinnitus as part of clinical history taking [43]. To ensure fairness across child and adult services, and to ensure that problematic tinnitus in children is not overlooked, the BSA Guidance recommends that all children attending audiology appointments are also routinely asked about tinnitus, and whether it is bothersome [14]. Where this approach has been adopted, paediatric tinnitus services have seen referral numbers steadily increase, indicating that this approach has enabled children to access needed care when they would not have done so previously [14].

### Tinnitus-related problems in children

A range of common problems experienced by children with troublesome tinnitus were identified suggesting that, like adults, children experience tinnitus-related problems that have a detrimental impact on their day-to-day life and wellbeing. The common problems identified by clinicians in this study; sleep difficulties, concentration difficulties, and emotional distress, were reflected in a recent review of children’s tinnitus-related problems reported in the literature [22]. Tinnitus problems relating to sleep, concentration, hearing, and psychological difficulties have also been reported as common in adult populations [2, 23, 24]. Unique to paediatric tinnitus, this evaluation highlighted children’s common experience of tinnitus-related concentration and hearing problems when at school or when studying. This suggests that consideration of the child’s school environment and educational demands are critical when assessing a child with tinnitus and deciding on an appropriate treatment strategy.

In this evaluation, a minority of respondents reported ‘lack of understanding of tinnitus’ or ‘lack of support from others’, as common experiences for children with tinnitus. Similar problem domains have shown fairly low prominence in adult studies. Watts et al. [23] found a minority of adults reporting a “need for knowledge” and Tyler and Baker [2] reported just one adult experiencing difficulty ‘explaining tinnitus to others’. Although, this could be due to Tyler and Baker’s participants feeling well informed and supported through their engagement in a self-help group [44]. Future research should establish whether ‘lack of understanding of tinnitus’ and ‘lack of support from others’ are particularly important issues for children with tinnitus. Unlike adults, children cannot easily access information to help them understand their tinnitus percept. Furthermore, children are limited in their ability to explain their difficulties to others around them [39]. To address the lack of child-friendly tinnitus resources, the BTA published a range of children’s information leaflets in and activity booklets [45].

### Limitations

Of approximately 200 NHS audiology services, this survey received responses from 87 departments, representing approximately 44%. Of those who did not respond, it is unknown how many offer a tinnitus service (adult only, or adult and paediatric). Thus, the results from this survey may not fully represent the population of UK paediatric tinnitus services. Furthermore, findings from this evaluation are UK-centric and specific to the nature of services within the NHS, and therefore may have limited relevance to paediatric tinnitus services based outside of the UK. Few studies outside of the UK have explored paediatric tinnitus service provision. Baguley et al. [9] studied data from expert centres in England, Germany, Poland, and Italy and found it was common for children to be seen within adult services as opposed to child-specific ones. Similarly, Rosing et al. [12] found children in Denmark were often seen in adult services. Investigators from both studies share the view that regardless of where children are managed, they should receive child-appropriate management from health care professionals with the appropriate skills and knowledge*.*

Finally, as the eight questions on paediatric tinnitus services were part of a larger service evaluation, they could only cover topics with limited depth. It will therefore be important to triangulate the findings from this evaluation with larger more in-depth research investigating these topics. One important topic that did not emerge from these data was the role of the parent. Particularly for younger children, parents are likely to be an essential source of information regarding the effect of tinnitus on the child’s life. It would therefore be valuable for future research to explore the role of the parent in tinnitus assessment and treatment and how the role of the parent can be harnessed to improve outcomes.

## Conclusion

Approaches used to manage children with troublesome tinnitus in UK NHS services are largely consistent and reflective of the current practice guidance. However, findings from this study indicate specialist staff training, access to child-specific tools, and the treatment and referral of children with tinnitus-related psychological problems represent key areas in need of optimisation. Children’s health care needs differ from those of adults and the tinnitus-related problems that are important to them are likely to differ from those considered important to adults. It is therefore essential for children to be managed using child-friendly approaches, by clinicians who are experienced in assessing and treating them. This warrants the development of effective, child-specific tinnitus services.

## Data Availability

The datasets used and/or analysed during the current study are available from the corresponding author on reasonable request.

## APPENDIX

1. Do you have a paediatric tinnitus service in your department?
  - Yes
  - No
2. Which members of your clinical team treat children with tinnitus (tick indicate all that apply)
  - Audiologist
  - Hearing therapist
  - Audiovestibular physician
  - Ear Nose and Throat doctor
  - Clinical psychologist
  - Psychiatrist
  - Other (please specify)
3. What does your paediatric tinnitus assessment include (tick all that apply)
  - History taking (tinnitus characteristics – descriptions of sounds
  - Annoyance/distress level
  - Mental health
  - Social/psychological impact (e.g. school performance)
  - Family history
  - Noise exposure history
  - Hearing difficulties
  - Other audiovestibular symptoms
  - Medical and Neurological history
  - Factors that affect the child’s tinnitus (e.g. external stresses)
  - The child’s current coping strategies
  - Other tests (please specify)
4. What measurement tools do you use to assess tinnitus related problems in children (tick all that apply)
  - A self-devised multi-item questionnaire measure of tinnitus related problems or distress
  - A Likert scale (a single line scale using descriptive categories of problem severity)
  - A visual analogue scale (single line scale without descriptive categories)
  - The Paediatric Index of Emotional Distress Questionnaire
  - The Strengths and Difficulties Questionnaire
  - The Revised Children’s Anxiety and Depression Scale
  - Questionnaires for educational assessment
  - Others (please specify)
5. What in your experience are the most common problems reported or identified in children who have bothersome tinnitus?
  - Open/ended response
6. What management options for tinnitus in children do you use in your department (tick all that apply)
  - Explanation, advice and information giving
  - Relaxation techniques
  - Mindfulness techniques
  - Sleep hygiene
  - Hearing aids
  - Wearable sound generators
  - Other sound enrichment
  - Cognitive Behaviour Therapy
  - Narrative Therapy
  - Other (please specify)
7. Approximately what percentage of children with tinnitus who present at your service require referral to Child and Adolescent Mental Health Services
  - Open/ended response
8. What (if anything) do you think could improve your paediatric tinnitus service?
  - Open/ended response

## Abbreviations

BSA: British Society of Audiology
BTA: British Tinnitus Association
CAMHS: Child and Adolescent Mental Health Services
CBT: Cognitive Behavioural Therapy
CHERRIES: Checklist for Reporting Results of Internet E-Surveys
ENT: Ear, Nose and Throat
Mini-TQ: Mini-Tinnitus Questionnaire
NHS: National Health Service
NIHR: National Institute for Health Research
PI-ED: The Paediatric Index of Emotional Distress Questionnaire
RCADS: Revised Children’s Anxiety and Depression Scale
SDQ: Strengths and Difficulties Questionnaire
TFI: Tinnitus Functional Index
THI: Tinnitus Handicap Inventory
UK: United Kingdom
